# VerificationTalk: A Verification and Security Mechanism for IoT Applications

**DOI:** 10.3390/s21227449

**Published:** 2021-11-09

**Authors:** Min-Zheng Shieh, Yi-Bing Lin, Yin-Jui Hsu

**Affiliations:** 1 Information Technology Service Center, National Yang Ming Chiao Tung University, Hsinchu 300093, Taiwan; 2 Department of Computer Science, National Yang Ming Chiao Tung University, Hsinchu 300093, Taiwan; liny@nctu.edu.tw; 3 Miin Wu School of Computing, National Cheng Kung University, Tainan 701401, Taiwan; 4 College of Humanities and Sciences, China Medicine University, Taichung 406040, Taiwan; 5 Department of Computer Science and Information Engineering, Asia University, Taichung 413305, Taiwan; 6 College of Artificial Intelligence and Green Energy, National Yang Ming Chiao Tung University, Hsinchu 300093, Taiwan; 7 Institute of Network Engineering, National Yang Ming Chiao Tung University, Hsinchu 300093, Taiwan; z8872453.cs08@nycu.edu.tw

**Keywords:** American Fuzzy Lop (AFL), bigraph models, formal methods, fuzz testing, Internet of Things (IoT)

## Abstract

An Internet of Things (IoT) application typically involves implementations in both the device domain and the network domain. In this two-domain environment, it is possible that application developers implement the wrong network functions and/or connect some IoT devices that should never be linked, which result in the execution of wrong operations on network functions. To resolve these issues, we propose the VerificationTalk mechanism to prevent inappropriate IoT application deployment. VerificationTalk consists of two subsystems: the BigraphTalk subsystem which verifies IoT device configuration; and AFLtalk which validates the network functions. VerificationTalk provides mechanisms to conduct online anomaly detection by using a runtime monitor and offline by using American Fuzzy Lop (AFL). The runtime monitor is capable of intercepting potentially harmful data targeting IoT devices. When VerificationTalk detects errors, it provides feedback for debugging. VerificationTalk also assists in building secure IoT applications by identifying security loopholes in network applications. By the appropriate design of the IoTtalk execution engine, the testing capacity of AFLtalk is three times that of traditional AFL approaches.

## 1. Introduction

In recent years, Internet of Things (IoT) applications have rapidly expanded [1] and new IoT applications have been deployed in many sectors including those of the smart city, smart medicine, smart agriculture, smart art and so on [2]. IoT application creation can be challenging and often relies on detailed knowledge of low-level protocols used for machine-to-machine communications. Therefore, several device integration and management platforms [3,4,5,6,7,8,9,10] have been proposed to hide the details of low-level protocols for IoT application development. In these platforms, an IoT application typically involves implementations in both the device domain and the network domain with flexibility. However, security issues come with the flexibility and diversity of IoT applications. Tudosa et al. [11] proposed the concept of the IoT trust pyramid to classify the IoT security issues into four layers: software security, hardware security, system security, and organization security. Software security is an idea to prevent crashes or other failures of IoT applications from malicious attacks and hacker risks. System security covers all aspects of data accessing and the integrity of IoT systems. Organization security and hardware security are out of the scope of this paper.

We focus on the verification and security issues of IoT device integration and management platforms. They play important roles in software security and system security. Platforms such as ThingsBoard [3], OpenMTC (commercial version of oneM2M) [5], CHT IoT Platform [6], Google Cloud IoT [7], AWS IoT [8], Microsoft Azure IoT [9] and Advantech WISE PaaS [10] provide very good API or SDK for IoT application development. These platforms also provide security tools such as the AWS IoT Device Defender, which continuously audits the configurations of IoT devices to improve their system security. Unfortunately, they do not provide verification and software security tools to guarantee the quality of the application codes developed using their API/SDK. Therefore, it is possible that application developers connect some IoT devices that should never be linked, perform the wrong operations on network functions, and implement the wrong network functions. These kinds of mistakes make IoT applications insecure in terms of their software. To resolve these software security problems, we propose the VerificationTalk mechanism to prevent inappropriate IoT deployment. VerificationTalk consists of two subsystems:

- BigraphTalk, which verifies IoT device configuration; and
- AFLtalk, which validates the network functions.

Based on BigraphTalk, VerificationTalk provides a runtime monitor to conduct anomaly detection in real time. The runtime monitor intercepts invalid data transmission to achieve system security. AFLtalk uses American Fuzzy Lop (AFL) [12] to identify software security issues in network applications. When VerificationTalk detects the errors, it provides feedback for debugging. To accelerate the execution of AFL, we specifically designed an appropriate IoT application development platform called IoTtalk. The paper is organized as follows. Section 2 overviews the related work; Section 3 proposes the IoTtalk/VerificationTalk architecture; Section 4 describes BigraphTalk and the Runtime Monitor; and Section 5 elaborates on AFLtalk.

## 2. Related Work

In this section, we introduce the tools related to the verification of IoT applications. Formal methods [13] are mathematically rigorous techniques for the specification, development, and verification of systems. Formal verification uses software tools to formally specify the properties of a system and verify whether the system implementation satisfies its specification. Tools based on formal methods (such as UML [14]) have basic support to express the safe connection of components. Among them, bigraphs are an expressive computational model, which provide better readability and ease of model extension through an intuitive graphical notation. Bigraphs [15] is a universal mathematical model for representing the spatial configuration of physical or virtual objects, their interaction capabilities, and temporal evolution [16]. A bigraph is a pair of relations over the same set of nodes:

- A directed forest, called a place graph, which represents the topological space in terms of node containment; and
- A hypergraph, called a link graph, which represents the interactions and non-spatial relations among nodes [16].

The system based on bigraphs is called the Bigraphical Reactive System, a universal formalism for modeling interacting systems that evolve in time and space. Bigraph has been applied to model a wide range of systems such as IoT/Edge systems [16] and Mixed Reality systems [17]. This paper proposes VerificationTalk which develops BigraphTalk to verify the connections among IoT devices by building their bigraphs. More related work on bigraphs and BigraphTalk can be found in [18] and the references therein.

In the network domain of an IoT application, we need to check whether a network function terminates for all inputs, which is an example of undecidable problems [19]. Such issues cannot be solved by formal methods. VerificationTalk utilizes an efficient software-testing technology called fuzz testing to resolve this issue. Fuzz testing is one of the most effective testing techniques to deal with correctness and security issues in software systems [20] such as memory corruption vulnerabilities and numeric errors. Fuzz testing traces a program with as many paths as possible to determine whether some inputs will crash or hang the program. In the past, testers have found many errors in accuracy and security risks in widely used software [21,22]. Fuzz testing has been adopted to detect the vulnerabilities in IoT devices [23,24] and IoT applications [25]. Since IoT devices usually have resource limitations and are prone to compromise, using fuzz tests to expose bugs in IoT applications is an effective way of removing program vulnerabilities.

## 3. The IoTtalk/VerificationTalk Architecture

**Figure 1:**
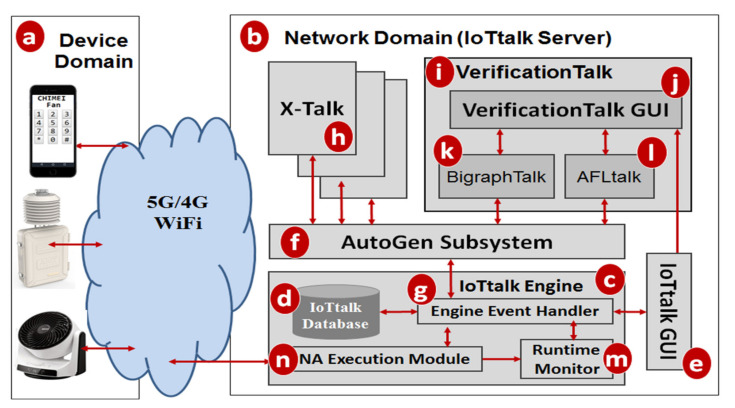
The IoTtalk and VerificationTalk architecture.

VerificationTalk was designed as the verification mechanism for IoTtalk [4], an IoT platform supporting the rapid development of network applications to drive the connected sensors and actuators. IoTtalk is defined in both the device and the network domains [4]. In the device domain (Figure 1a), an IoTtalk device installs a device application to connect to the IoTtalk server in the network domain (Figure 1b). Device application is out of the scope of this paper and the reader is referred to [4] for further details.

In the network domain, the IoTtalk server is responsible for provisioning network applications (NAs) that manipulate the connections and meaningful interactions among IoT devices. An IoTtalk application is a set of NAs. The server consists of several subsystems. The IoTtalk Engine (Figure 1c) systematically creates and manages the NAs for connecting the IoT devices, and is responsible for the NA execution to provide the end-to-end communications between the IoTtalk devices and the server.

**Figure 2:**
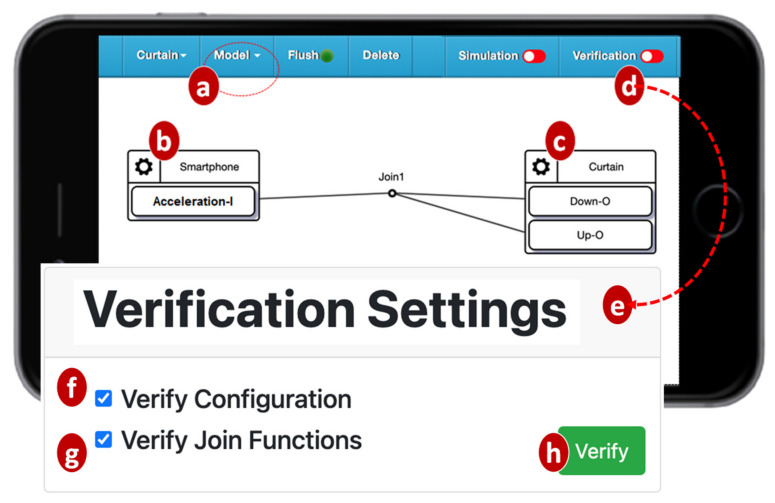
Configuring an IoT application using the IoTtalk GUI.

The IoTtalk Engine stores information such as the IoT devices and their connections in the IoTtalk Database (IoTtalk DB; Figure 1d). The IoTtalk Graphical User Interface (GUI, Figure 1e) is a friendly web-based user interface that allows a developer to create network applications by connecting the IoT devices and efficiently writing the NAs. Through the IoTtalk GUI, the AutoGen subsystem (Figure 1f) automatically generates the NAs and the integration of NAs for specific IoTtalk applications called X-Talk (Figure 1h). Examples of X-Talk include AgriTalk [26], PigTalk [27] and PuppetTalk [28]. Details of AutoGen can be found in [29]. Figure 2 illustrates a simple IoTtalk GUI example where a smartphone controls a curtain. This application attempts to move up (down) the curtain when the smartphone faces up (down). By selecting the smartphone and the curtain from the Model pulldown list (Figure 2a), the GUI shows the smartphone icon (Figure 2b) and the curtain icon (Figure 2c). We use the input device feature (IDF), i.e., the Acceleration-I sensor of the smartphone, to control the output device features (ODFs; i.e., Up-O and Down-O buttons) by dragging the Join 1 line to connect the IDF to the ODFs.

The VerificationTalk Subsystem (Figure 1i) is one of the X-Talk applications supported by AutoGen. Specifically, the VerificationTalk HTTP Service procedure in AutoGen is used to retrieve the information about the IoT application to be verified—including the details of device configurations and NAs of the application. The VerificationTalk Subsystem consists of two components: BigraphTalk (Figure 1k) and AFLtalk (Figure 1l). VerificationTalk is associated with a web-based GUI (Figure 1j). By clicking the Verification button (Figure 2d), the IoTtalk GUI will jump to the VerificationTalk GUI (Figure 2e). This GUI allows the developer to select the BigraphTalk or the AFLtalk mechanisms (Figure 2f,g) to be executed and specify forbidden configurations to be elaborated later. 

Two major network protocols are used in IoTtalk: Message Queueing Telemetry Transport (MQTT) for links (a)–(n), and (g)–(f); and HyperText Transfer Protocol Secure (HTTPS) for links (g)–(e), (f)–(h), (f)–(k), (f)–(l), and (e)–(j). The IoTtalk DB interacts with the IoTtalk Engine through the link (g)–(d) using the Object Relational Mapping (ORM) protocol [30]. 

In the subsequent sections, we will elaborate on BigraphTalk (Figure 1k) and AFLtalk (Figure 1l).

## 4. BigraphTalk

**Figure 3:**
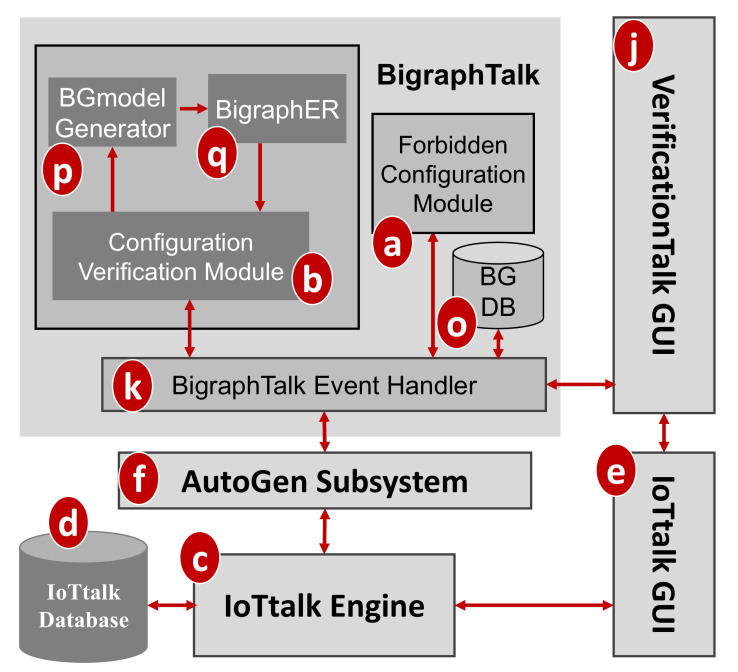
BigraphTalk architecture.

BigraphTalk (Figure 1k) consists of three major components (Figure 3a,b,o) controlled by the BigraphTalk Event Handler (Figure 3k). The Forbidden Configuration Module (Figure 3a) manages the records of forbidden configurations stored in the Bigraph Database (BG DB; Figure 3o). The Configuration Verification Module (Figure 3b) composes the messages for configuration verification requests and interprets the results after verifying the configuration of the IoTtalk application.

To specify a forbidden configuration, a domain expert manipulates the related devices and device features through the VerificationTalk GUI (Figure 1j) which instructs the Forbidden Configuration Module (Figure 3a) to create, modify, or delete the tables related to the forbidden configuration. This set of forbidden configuration tables automatically applies to all IoT applications that have this configuration. This way, BigraphTalk guarantees the configuration correctness of IoTtalk applications even if the application developers have no domain knowledge about configuration correctness.

To verify an IoT application, the developer clicks the Verification button in the IoTtalk GUI (Figure 2d) and the page will jump from the IoTtalk GUI to the VerificationTalk GUI (Figure 2e). The developer then selects the verification mechanisms. Suppose that “Verify Configuration” is chosen (Figure 2f); the GUI then sends the request to the Configuration Verification Module through the BigraphTalk Event Handler (Figure 3k). The Configuration Verification Module provides the configuration information of the IoTtalk application to the Model Generator (Figure 3p) to construct an instance of the bigraph model corresponding to the target IoTtalk application. Then, BigraphER (Figure 2q) [31] validates the model by matching the model against the forbidden configuration and sends the result back to the Configuration Verification Module. The Model Generator retrieves the target application information from the IoTtalk Database (Figure 1d) through the VerificationTalk HTTP Service procedures of the AutoGen Subsystem (Figure 1f) and displays the result of verification on the IoTtalk GUI (Figure 1e).

BigraphER is a suite of open source tools that provide the specification and verification of bigraphs. An advantage of BigraphER is its provision of a library of matching routines. BigraphER is the only tool supporting features such as parameterized entities essential for our implementation.

### 4.1. Integrating Bigraph with IoTtalk

**Figure 4:**
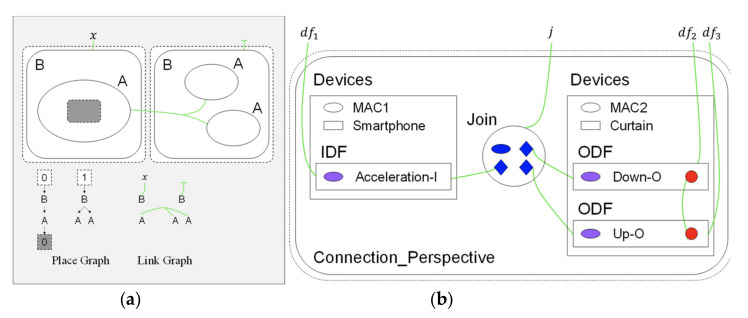
Bigraph representations: (**a**) basic concept; and (**b**) bigraph for “curtain control by smartphone”.

A bigraph is composed of a pair of relations over the same set of nodes: the place graph specifies spatial relationships while the link graph specifies the non-spatial interactions [32]. A place graph represents the locality by node placing. A link graph encodes the connectivity by the hyperlinks between entities. Entities, real or virtual, are encoded by nodes, represented by ovals or circles. Nodes are assigned a type, called control, such as labels A and B in Figure 4a. The locality between entities can be spatially illustrated by nesting one entity within or alongside another. A region represented by a dashed rectangle specifies the adjacent part of the system. Shaded rectangles are called sites that encode components of the system that have been abstracted away. Connectivity is defined by the green hyperlinks that connect the entity to the name (such as “x”), the other entities, or do not connect to anything. A hyperlink may connect multiple entities, such as all “A” entities. Each entity is assigned an arity that determines its number of links. Similar entities always have the same number of links.

The BigraphTalk defines encoding from the IoTtalk applications to specific instantiations of bigraph models for the correctness examination of applications. The encoding uses bigraph models to mimic the connection perspective of IoTtalk applications. If any forbidden configuration exists, BigraphTalk can detect them from the corresponding bigraph models. The mapping between IoTtalk components and bigraph entities are described in [18], where each bigraph entity has a fixed arity that determines the number of links, a contained-by relation that defines its placement, and the entities can link with it. By using these entities and the message retrieved from the database of the IoTtalk engine (Figure 1d), the BGmodel Generator (Figure 3p) constructs the bigraph instance corresponding to the IoTtalk application. The message from the IoTtalk engine includes details of the devices, Joins, and connections in the JSON format. The bigraph models that represent the connection perspective of Figure 2 are shown in Figure 4b.

**Figure 5:**
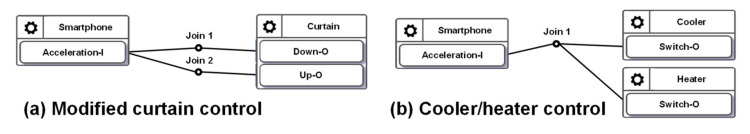
(**a**) A potential forbidden configuration; and (**b**) a forbidden configuration.

In Figure 2, when the values sent from the IDF reach the Join 1 point, the IoTtalk server computes the new values for ODFs connected to this Join point. Each ODF connected to the same Join point will receive the same value. However, in the curtain control application, we should not send the same values to control the Up–O and the Down–O buttons at the same time. Therefore, the configuration in Figure 2 is considered a forbidden configuration. BigraphTalk will give an error message when the IDF and the ODFs are connected through Join 1. The developer resolves this issue by connecting the IDF to the ODFs with individual Join links where Join 2 is an inverse function (Figure 5a). Another forbidden configuration is illustrated in Figure 5b, where both the cooler and the heater in the same room are turned on when the smartphone faces up.

**Figure 6:**
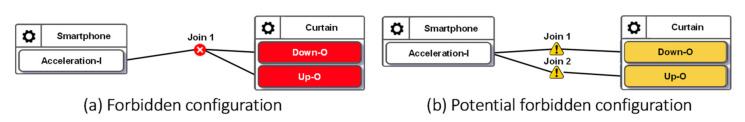
Alerts for forbidden and potential forbidden configurations.

The forbidden configurations can be statically detected before execution as they occur using the links between the devices that should never have any connection. The IoTtalk GUI alerts the developer of invalid configurations. If the verification results indicate a forbidden configuration, the erroneous device features and Joins are colored in red (see Figure 6a). IoTtalk GUI also warns the users of potential errors by coloring the corresponding device features and Joins yellow (see Figure 6b).

**Figure 7:**
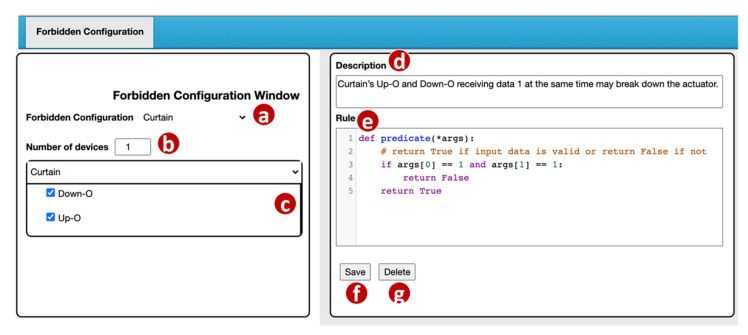
Forbidden configuration management window.

The VerificationTalk GUI provides a Forbidden Configuration Management window (Figure 7) for a domain expert to manage the forbidden configurations. In the Forbidden Configuration pulldown list (Figure 7a), the domain expert selects an existing configuration to modify or create a new configuration.

For a new configuration, the domain expert first specifies the number of devices included in this configuration (Figure 7b). For each device, BigraphTalk (Figure 1k) retrieves its device features from the IoTtalk Database (Figure 1d). These device features are listed in the Curtain window (Figure 7c) and are selected by the domain expert if they are involved in the forbidden configuration. If the selected device features within a device connect to the same input, it will be detected as an error. For example, BigraphTalk detects the forbidden configuration in Figure 2, where the curtain will simultaneously move up and down.

When the error is detected, an error message is displayed, which is specified by the domain expert in the Description field (Figure 7d). The Rule field (Figure 7e) is a code editor that allows the domain expert to write a rule function “predicate” for the Runtime Monitor (Figure 1m) to determine whether the transmitted data are valid. In this predicate example, args[0] represents Down–O and args[1] represents Down–I. Details of the Runtime Monitor will be elaborated in Section 4.2. When the domain expert clicks the “Save” button (Figure 7f), the GUI invokes the Forbidden Configuration Module (Figure 3a) to create or update the forbidden configuration record in the BG DB (Figure 3o).

The BG DB maintains the forbidden configuration information in two tables: ForbiddenConfiguration and ForbiddenFeature. The ForbiddenConfiguration table maintains the forbidden configuration attributes including the description field (the error message in Figure 7d) and the rule field (the predicate in Figure 7e). A record in the ForbiddenFeature table stores the information of all device features in a forbidden configuration including the device name (Figure 7a) and the device features (Figure 7c).

The Forbidden Configuration Module (Figure 3a) provides two procedures for forbidden configuration operations. The procedure create_forbidden_configuration() creates a forbidden configuration when the “Save” button (Figure 7f) of the GUI is clicked. The procedure get_forbidden_configuration_info() returns the information of a forbidden configuration to the GUI when the configuration is selected in the Forbidden Configuration pulldown list (Figure 7a).

### 4.2. Runtime Monitor

Unlike the forbidden configurations, when BigraphTalk identifies a potential forbidden configuration (Figure 6b), the Runtime Monitor (Figure 1m) in the IoTtalk Engine inserts a runtime monitor process and a rule function provided by the Forbidden Configuration Module in the execution path of the configuration. In the normal execution mode, the data transmitted between the IoTtalk server and the IoT devices (Figure 1a) only need to go through the NA Execution module (Figure 1n). In the runtime monitor mode, the NA Execution Module will also pass the execution results to the Runtime Monitor (Figure 1m) to check whether it is invalid. 

**Figure 8:**
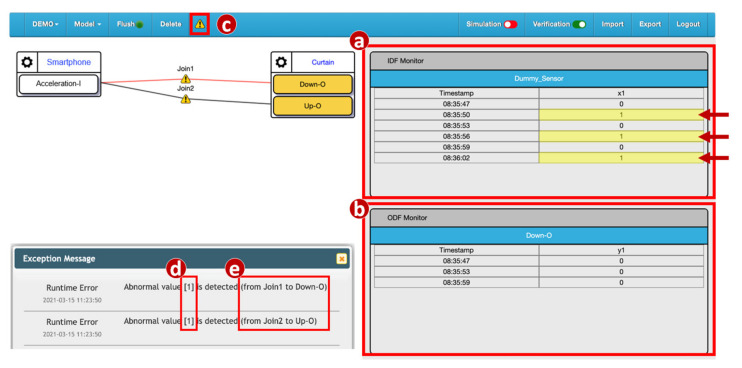
Execution of the Runtime Monitor process.

Before the data actually reach the output device, the Runtime Monitor process checks whether they are forbidden. If not, the output device receives the data normally. If so, the data are discarded. This mechanism attempts to detect quickly anomalies before any wrong configuration causes the device to break down. In terms of cyber security, the Runtime Monitor intercepts the harmful data and prevents damage to the IoT devices. The Process class from the multiprocessing module [33] is used to construct the Runtime Monitor process. Before the process is activated, the data transmitted from input devices to output devices go through the Join links and are processed at the NA Execution Module only (Figure 1n). The Runtime Monitor process is activated by clicking the alert icon (button) in the IoTtalk GUI (Figure 8c) and the data arriving at the Join points are examined by the Runtime Monitor using the rule specified in Figure 7e. In this example, the values “1” sent from the IDF Acceleration-I are displayed in the IDF Monitor (Figure 8a). The IDF data are then examined and modified by the rule. Then, the ODFs Up–O and Down–O of the curtain only receive the values 0. The ODF data are displayed in the ODF Monitor (Figure 8b), and the IoTtalk GUI pops up an error message dialog window to show the value of the invalid data (Figure 8d) and the segment where it occurred (Figure 8e).

**Figure 9:**
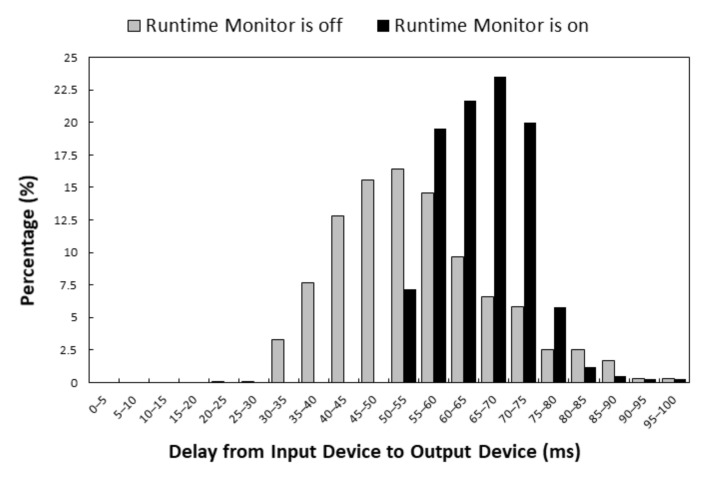
Histograms of delays when the Runtime Monitor is on/off.

We evaluated the execution time of the Runtime Monitor through measurement experiments. The experiments were conducted on the IoTtalk server and the devices were connected to the server through WiFi, 4G LTE or 5G. The IoTtalk server was equipped with an Intel Core i7 4770 (3.4 GHz), 16 GB RAM, and Ubuntu 16.04 was installed. Figure 9 illustrates the histograms of the execution delays from the input device to the output device when the Runtime Monitor was off (gray bars) and when it was on (black bars). When the Runtime Monitor was turned off, the delay included the transmission time from the input device to the IoTtalk server, the processing time at Joins, and the transmission time from the IoTtalk server to the output device. When the Runtime Monitor was turned on, the delay included an additional execution time of the runtime monitor process. We repeated the experiments 1000 times for each scenario. When the Runtime Monitor was off, the average delay was 54.62 milliseconds, and the standard deviation was 13.03 milliseconds. When the Runtime Monitor was on, the average delay was 65.42 milliseconds, and the standard deviation was 7.65 milliseconds. Our study indicates that the Runtime Monitor increased the expected delay by 20%, which is an acceptable overhead for debugging.

**Figure 10:**
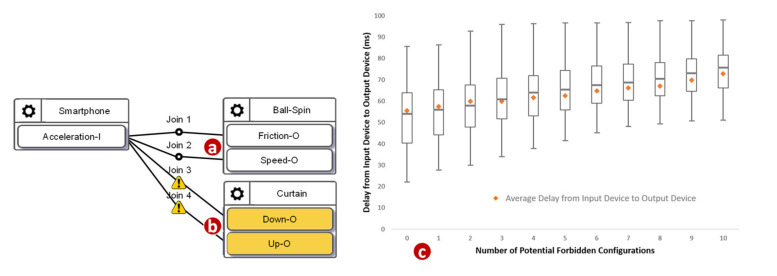
A normal configuration, a potential forbidden configuration, and the execution delays.

We then investigated how the number of potential forbidden configurations affects the delay. We used a synthetic application with ten configurations. Among these ten configurations, some of them were correct, such as the one illustrated in Figure 10a, and some of them were potential forbidden configurations, such as the one illustrated in Figure 10b. Figure 10c plots the minimums, the first quartiles, the medians, the third quartiles, and the maximums of 1000 delays against the number of the potential forbidden configurations (where the total number of configurations is 10). The delay roughly linearly increased as the number of potential forbidden configurations increased. When there was no potential forbidden configuration, the maximum among these 1000 delays was 85.8 milliseconds, the median was 54.08 milliseconds, the minimum was 22.25 milliseconds, and the average was 54.32 milliseconds. When the number of potential forbidden configurations was 10, the maximum among these 1000 delays was 98.2 milliseconds, the median was 75.6 milliseconds, the minimum was 51.2 milliseconds, and the average was 74.58 milliseconds. As the number of potential forbidden configurations increased, the interquartile range became narrower. The growth rate of the maximum was relatively low. Our study indicates that the execution overhead of the Runtime Monitor was acceptable.

## 5. AFLtalk

While BigraphTalk is effective for checking whether users connect some devices that should never have any connection, it cannot check whether a program terminates for all inputs. To resolve this undecidable problem [19] of the NAs (join functions), VerificationTalk utilizes a security-oriented fuzzer called American Fuzzy Lop (AFL) [12]. The AFL provides large functional coverage for the fuzzed code by employing compile-time instrumentation and genetic algorithms to automatically identify test cases to trigger new internal states in the binary target. Previous studies [34,35] have shown that the extension of AFL has been successfully applied to the field of IoT and can efficiently and automatically find vulnerabilities in real-world IoT programs.

**Figure 11:**
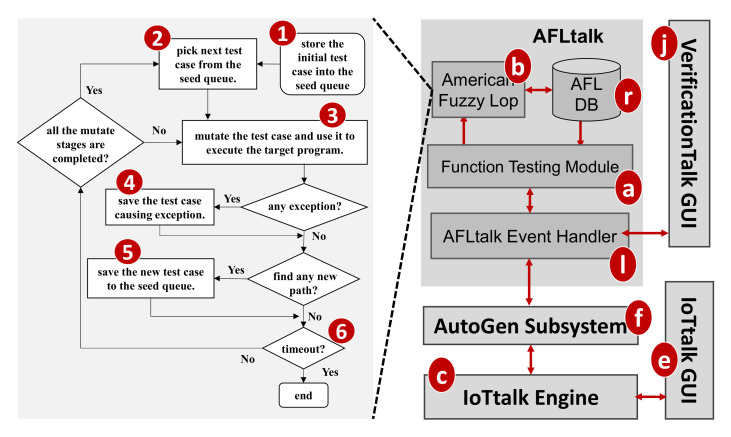
The AFLtalk architecture.

**Figure 12:**
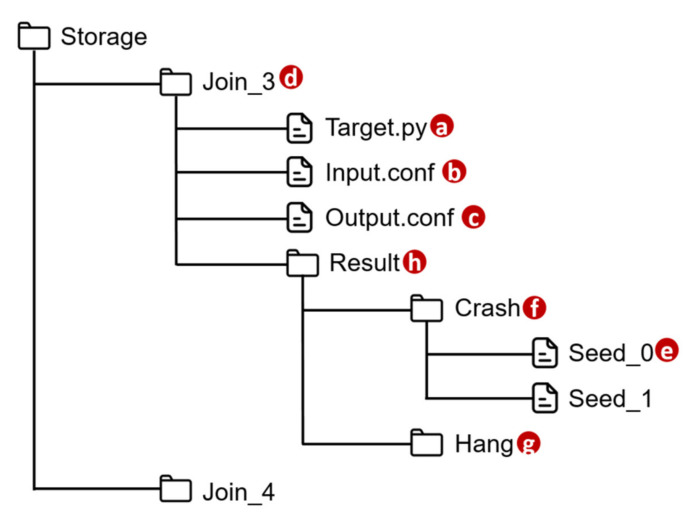
Directory structure of AFLtalk DB.

VerificationTalk implements AFLtalk (Figure 1l), which consists of the following components. The Function Testing Module (Figure 11a) collects the information related to the Join functions to be tested, decodes the results after verifying the target Join functions, stores the target function details into the AFLtalk DB (Figure 11r) and instructs AFL (Figure 11b) [12] to conduct fuzz testing. The AFL completes the testing, and stores the testing results into the AFLtalk DB. When the Function Testing Module receives the request for Join functions testing from the Verification GUI (Figure 11j), the following actions are taken. First, the Function Testing Module retrieves the information of the Join functions from the IoTtalk engine (Figure 11c). The information includes the target code and the data type/range of the input/output. The Function Testing Module stores the target code into the file of AFLtalk DB named Target.py (Figure 12a). The DB also stores the details of the inputs and outputs in Input.conf (Figure 12b) and Output.conf (Figure 12c), respectively. These three files will be stored under a specific directory (Figure 12d) with the same name as the corresponding Join. Then, the AFL loads the target functions with inputs and outputs from these three files and initializes the fuzz testing.

Once the AFL has completed fuzz testing, it will obtain the inputs causing an error in the target function and store each of these inputs into the file (Figure 12e) under the Crash directory (Figure 12f) or Hang directory (Figure 12g) according to their error type. These two directories are organized under the Result directory (Figure 12h). To assist in debugging the IoTtalk applications, the Function Testing Module extracts the identifiers of erroneous Joins and the corresponding inputs from the verification results. Then, the IoTtalk Engine pushes the information to the IoTtalk GUI (Figure 11e) for display.

As a security-oriented fuzzer, the AFL employs a novel type of compile-time instrumentation and genetic algorithms to automatically discover test cases that trigger new internal states in the targeted binary. This approach substantially improves the functional coverage for the fuzzed code. The AFL works as follows. It first stores the user-supplied initial test cases into the seed queue (Figure 11 (1)). Then, it takes the next input file from the seed queue and trims the test case to the smallest size without changing the measured behavior of the Join function under test (Figure 11 (2)). The AFL uses this test case to execute the target program (Figure 11 (3)). Before execution, the AFL mutates the input file, repeatedly using various fuzzing strategies such as bit flipping. If the execution causes any exceptions, the AFL saves the test cases resulting in the exceptions (Figure 11 (4)). The exception for a kernel panic or a fatal system error can be divided into two categories: crash and hang. A crash is an event that causes the tested program to receive a fatal signal. On the other hand, a hang occurs when the tested program ceases to respond to inputs. For example, the program never returns if it has any infinite loops. The AFL adds a new mutated output entry to its seed queue if the mutation causes a new state transition recorded by the instrumentation (Figure 11 (5)). Then, the AFL takes the next input file from the seed queue and repeats the process until the preset timer expires (Figure 11 (6)).

Since the Join functions are written in Python, AFLtalk modifies the extension Python AFL [36] as a fuzzer. The modification allows AFLtalk to specify the data type/range of the input/output to discover more potential errors, such as “the returned value is out of range”. For example, we design an application that can control the rotation angles of a robot hand through the outputs of a specific program. Since the rotation angles of the robot hand have the limitation, if the outputs of the program exceed their tolerable ranges, the motors of the robot hand may be damaged. AFLtalk can discover such errors in advance. Another feature of AFLtalk is that it can replace unit testing. Since the test cases of the target program are randomly generated, AFLtalk supports automated testing so that the developers do not need to design test cases by themselves.

### 5.1. Join Function Verification

**Figure 13:**
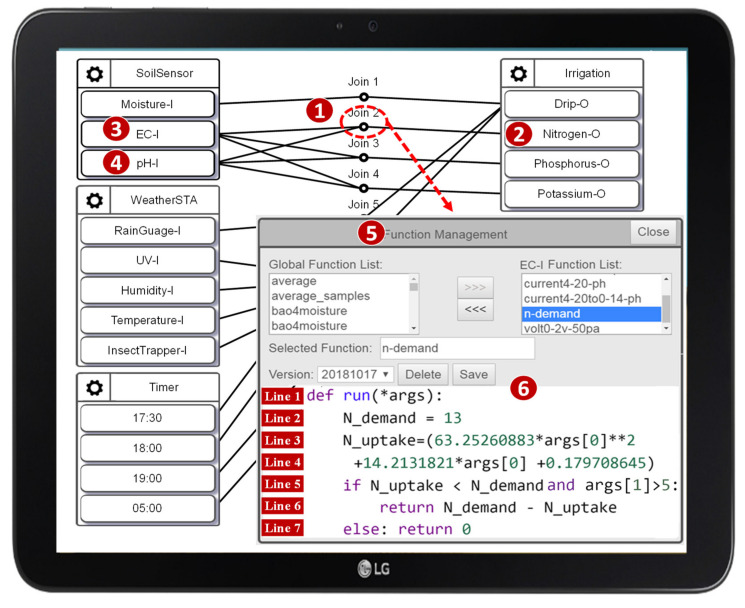
The AgriTalk configuration [26].

An IoTtalk NA is implemented as a Python Join function, which performs data transformation and decision logic on the data transmitted between IoT devices. For example, the IoTtalk application in Figure 13 automates the irrigation, fertilization, and pest control process, which improves crop cultivation [26] through the interaction between the sensors, the controllers, and the actuators. The Join functions realize automatic operations between the actuators and the controllers. Consider the n-demand function executed at Join 2 (Figure 13 (1)), which estimates the addition of nitrogen fertilizer (Figure 13 (2)) controlled by the values sent from EC and pH sensors (Figure 13 (3) and (4)). By clicking the Join 2 circle, the Join Function Management window pops up (Figure 13 (5)). The Python code for the Join function n-demand is written in Figure 13 (6).

**Figure 14:**
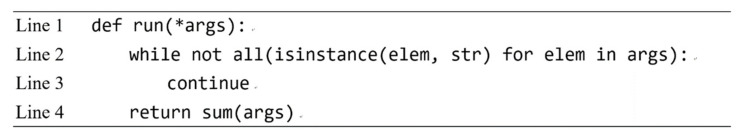
An example of the erroneous Join function.

In Figure 13 (6), Line 1 defines the input argument args of the Python function run(). The argument args stores the EC (args[0]) and the pH (args[1]) values of the soil sent from IDFs EC-I and pH-I, respectively. Line 2 sets the demand for nitrogen fertilizer. Lines 3 and 4 compute the amount of nitrogen fertilizer in the soil using the equation defined in [26]. Lines 5–7 determine the addition of nitrogen fertilizer. In this example, the Join function passes the AFLtalk test. As another example, Figure 14 gives an erroneous Join function. Line 2 checks whether the data types of the arguments passing to this Join function are all strings. If the data type of any argument is not a string, this while loop will become an infinite loop, which will cause a hang error. Otherwise, Line 4 will perform the sum() method on the arguments whose data types are all strings, which will cause a crash error.

### 5.2. Time Complexity of AFLtalk

**Figure 15:**
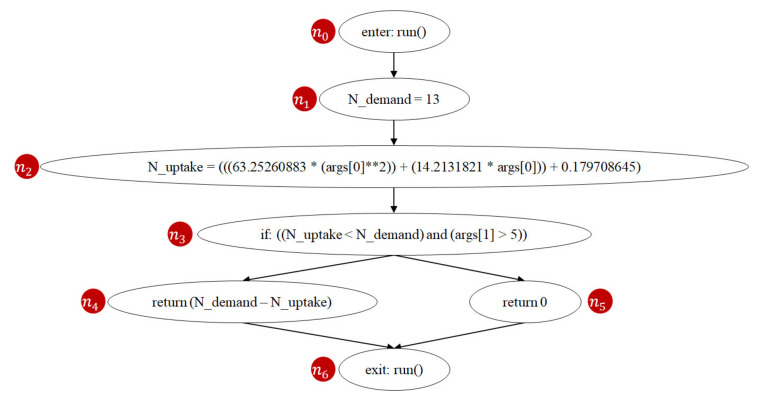
Control-flow graph of the Join function n-demand.

AFLtalk utilizes control-flow graphs [37,38] to analyze the complexity of the program to be tested. A control-flow graph is a graphical representation of all paths that might be traversed through a program during its execution [39]. Figure 15 shows an instance of the control-flow graph of the n-demand function. In this graph, a node represents a basic block (i.e., a straight-line code sequence without any branches), and each directed edge represents a branch. There are two specific nodes: the entry node (Figure 15 (*n*_0_)) represents the entry point into the flow graph, while the exit node (Figure 15 (*n*_6_)) represents the control flow exit.

When a test suite runs a program, code coverage [40,41] is used to measure the degree of execution of that program. Several coverage criteria were introduced and compared in [42], such as node coverage, edge coverage, etc. The higher the code coverage, the more executed portions of the source code under test, and therefore the lower the chance of containing undetected software bugs. Since code coverage is a moderate indicator for the capability of fault detection, full code coverage may imply that all potential bugs can be detected [43]. Full code coverage time is the delay for the fuzzer to achieve the full code coverage of the program, which measures the quality of the coverage-guided fuzzing engines (e.g., AFL, libFuzzer, and Honggfuzz) [44].

Prime path [40,42] is a coverage criterion used in software testing. A path from node *n_i_* to node *n_j_* is a prime path if it is simple [45] and does not appear as a subpath of any other simple path. A simple path is a path that does not contain repeated nodes. For example, Figure 15 shows that the n-demand function has two prime paths (*n*_0_, *n*_1_, *n*_2_, *n*_3_, *n*_4_, *n*_6_) and (*n*_0_, *n*_1_, *n*_2_, *n*_3_, *n*_5_, *n*_6_). Prime path coverage indicates the degree of the executed prime paths. Previous studies [40,42] show that prime path coverage provides a better quality of coverage as it subsumes node coverage and edge coverage. If we follow the prime path coverage criterion, achieving full code coverage means that all prime paths of the program have been executed. Since the number of prime paths is positively correlated to software complexity [46], the developers dedicate more efforts testing when the number of prime paths of the program increases.

Clearly, changing the number of prime paths will inevitably affect the time taken to achieve full code coverage. To see the impact of prime paths, we design a set of synthetic join functions based on [47], and their prime paths vary from 1 to 16. We designed these synthetic join functions based on the functions provided by the machine learning-related projects (e.g., scikit-learn [48]) because they are similar to the operations of the IoTtalk Join functions (e.g., data transformation and decision logic). For each synthetic Join function, we measure 100 full code coverage times in AFLtalk for analysis.

Table 1 lists the average full code coverage times measured from AFLtalk. The average full code coverage time is 1.119 s when there are two prime paths, 2.269 s when there are four prime paths, 8.194 s when there are eight prime paths, and 30.787 s when there are sixteen prime paths. As the number of prime paths grows, the average full code coverage time significantly increases.

**Figure 16:**
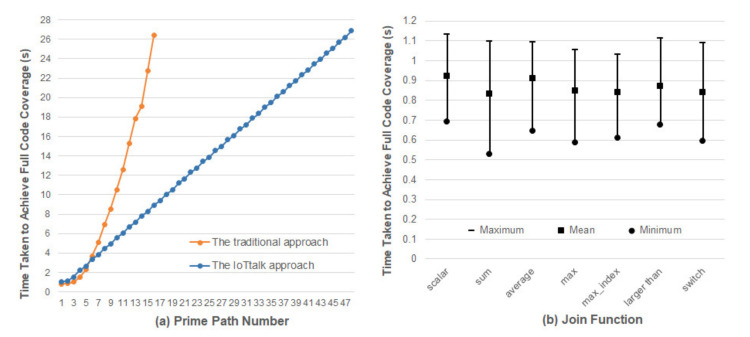
Comparison between the traditional one-piece NA approach and the IoTtalk Join-function approach.

In traditional IoT platforms such as [3,5], an IoT application is implemented as a network program in the network domain which may have many prime paths, and Table 1 indicates that significant efforts are required to discover their potential errors. To reduce the full code coverage time, IoTtalk uses the divide-and-conquer approach that partitions a big network program into several NAs or Join functions [4]. A Join function involves simple connections between a small number of IDFs and ODFs. Therefore, the number of prime paths in a Join function is typically no more than 3. The IoTtalk Engine is a global event handler that dispatches the event to the corresponding Join functions. The general mechanism of the IoTtalk Engine has been verified to be error-free. To ensure code coverage for an IoTtalk application, we only need to handle the prim paths of the Join functions. That is, by translating the original one-piece IoT application to multiple modularized Join functions in IoTtalk, the application with a large prime path number can be split into multiple Join functions with small prime path numbers (i.e., one, two or three prime paths) for testing. For instance, a complex application with sixteen prime paths can be split into eight Join functions, each with two prime paths. In IoTtalk, we can separately perform fuzz testing on these Join functions with small prime paths to reduce the time taken to achieve full code coverage. Figure 16a shows that the full code coverage time of an application with sixteen prime paths will be reduced from 26.39 s to 8.95 s. Therefore, the IoTtalk approach can greatly reduce the time required to find potential errors in IoT applications. The figure also indicates that with a time budget of 30 s, the traditional approach only finds potential errors in the IoT applications with 16 prime paths. On the other hand, the IoTtalk approach finds potential errors in the IoT applications with 48 prime paths within the same amount of time.

Since the initial seed of the program is randomly generated by AFLtalk, we should investigate whether the initial seed will affect the full code coverage time. We collected a set of widely used Join functions introduced in [4]. For each Join function, we performed fuzz testing 1000 times to measure the full code coverage times. Figure 16b plots the maximums, the means, and the minimums among all measured full code coverage times, respectively. From the ratio of the maximum to the mean, we observe the effects of initial seeds on the full code coverage times, which are different for various Join functions. The worst case occurs in the sum function where the expected full code coverage time is 0.834 s, and the maximum time is 31.76% higher than the average time. The best case occurs in the Join function “average” where the expected time is 0.911 s, and the maximum time is 19.9% higher than the average time. The impact of the initial seed may increase the execution overhead by 30%, which is acceptable for AFTtalk.

## 6. Conclusions

This paper proposed the VerificationTalk mechanism to prevent inappropriate IoT application deployment. VerificationTalk consists of two subsystems: BigraphTalk verifies IoT device configuration and AFLtalk validates the network functions. VerificationTalk conducts online anomaly detection by using a runtime monitor and offline by using AFL. When VerificationTalk detects the errors, it provides the feedback to the developers for debugging. By the appropriate design of the IoTalk engine, the execution capacity of AFL is three times that of traditional approaches. 

There are two future directions for VerificationTalk:

- BigraphER supports parameterized entities that can be used to model device location. Such property can be used in BigraphTalk to enhance the definition of forbidden configuration. For example, some chemical substances should not be placed near a factory. With models of device location, VerificationTalk can support smart agriculture applications such as [49] better.
- BigraphTalk will provide the property of the device ownership. For instance, only the residents may access the data within their dormitory. We can ensure users’ privacy in intelligent medical applications similar to [50] with ownership properties.

## Figures and Tables

**Table 1:** Full code coverage time against the number of prime paths.

Prime Path Number	1	2	3	4	5	6	7	8
Average Full Code Coverage Time (s)	1.06	1.12	1.55	2.27	3.19	4.66	6.55	8.19
Prime Path Number	9	10	11	12	13	14	15	16
Average Full Code Coverage Time (s)	9.88	12.06	14.39	17.37	20.18	22.83	26.72	30.79

## Data Availability

Publicly available datasets were analyzed in this study. This data can be found here: [https://github.com/mzshieh/verificationtalk-exp-data].
